# Evaluation of postoperative surveillance strategies for esophago-gastric cancers in the UK and Ireland

**DOI:** 10.1093/dote/doab057

**Published:** 2021-08-24

**Authors:** Swathikan Chidambaram, Viknesh Sounderajah, Nick Maynard, Tim Underwood, Sheraz R Markar

**Affiliations:** Department of Surgery and Cancer, Imperial College London, London, UK; Department of Surgery and Cancer, Imperial College London, London, UK; Department of Surgery, Churchill Hospital, Oxford University Hospitals NHS Trust, UK; Department of Gastrointestinal Surgery, University of Southampton, UK; Department of Surgery, Churchill Hospital, Oxford University Hospitals NHS Trust, UK; Department of Molecular Medicine and Surgery, Karolinska Institutet, Stockholm, Sweden; Department of Surgery and Cancer, Imperial College London, London, UK

**Keywords:** esophageal cancer, gastric cancer, recurrence, surveillance

## Abstract

Esophago-gastric malignancies are associated with a high recurrence rate; yet there is a lack of evidence to inform guidelines for the standardization and structure of postoperative surveillance after curatively intended treatment. This study aimed to capture the variation in postoperative surveillance strategies across the UK and Ireland, and enquire the opinions and beliefs around surveillance from practicing clinicians. A web-based survey consisting of 40 questions was sent to surgeons or allied health professionals performing or involved in surgical care for esophago-gastric cancers at high-volume centers in the UK. Respondents from each center completed the survey on what best represented their center. The first section of the survey evaluated the timing and components of follow-ups, and their variation between centers. The second section evaluated respondents perspective on how surveillance can be structured.

Thirty-five respondents from 27 centers consisting 28 consultants, 6 senior trainees and 1 specialist nurse had completed the questionnaire; 45.7% of responders arranged clinical follow-up at 2–4 weeks. Twenty responders had a specific postoperative surveillance protocol for their patients. Of these, 31.4% had a standardized protocol for all patients, while 25.7% tailored it to patient needs. Patient preference, comorbidities and chance of recurrence were considered as major factors for necessitating more intense surveillance than currently practiced. There is a significant variation in how patients are monitored after surgery between centers in the UK. Randomized controlled trials are necessary to link surveillance strategies to both survival outcomes and quality of life of patients and to evaluate the prognostic value of different postoperative surveillance strategies.

## INTRODUCTION

Esophageal and gastric cancers aggressive malignancies with a high recurrence rate even after treatment with curative intent. In the past decade, the prognosis has improved due to better pre-operative staging investigations as well as dramatic progress in the surgical and non-surgical management of these patients.^1^^,^^2^ Nevertheless, although in-hospital postoperative mortality rate is below 5% in the UK, it does not always translate to better long-term survival outcomes.^3^ Mariette et al. reported that over 50% of recurrent disease is evident in the first 2 years after curatively intended surgery. Regular follow-up after definitive treatment is a crucial part of postoperative management in order to diagnose and treat benign complications of cancer treatment; identify recurrent or metastatic disease; assess and manage nutritional disorders; and provide the necessary psychosocial support to patients.^4^^,^^5^ The prognostic benefit of standardized surveillance protocols has been previously studied in other gastrointestinal malignancies with effective curative treatments and provides further support for extending this research to esophageal and gastric cancers.^6–8^

Previous studies have evaluated a variety of surveillance strategies, involving regular clinical assessment, cross-sectional imaging, and endoscopy, or a pathway relying on symptoms to trigger further follow-up investigations.^4^^,^^9^^,^^10^ Previous studies have shown disparate results regarding the prognostic implications of an intensive radiological or biomarker-based follow-up protocol over a symptom-based follow-up strategy. This is reflected by the wide international variation on guidelines for follow-up.^11^ For example, high-intensity surveillance involving computed tomography (CT) imaging and endoscopy is prevalent in countries like Japan, while minimal in most European centers.^12–14^ In the UK, the National Institute for Clinical Excellence (NICE) guidelines do not recommend routine clinical follow-up or radiological investigations in the absence of symptoms, however they do identify this as one of the important areas for future research.^15^ Hence, there is a paucity of high-quality evidence on how postoperative surveillance should be carried out for this cohort of patients, who may highly benefit from it. Thus, the primary objective of this survey-based study was to characterize the current pattern of postoperative surveillance amongst high-volume centers carrying out esophago-gastric resections in the UK, and to summarize the current opinions of how this can be changed to improve the patient experience and survival outcomes.

## METHODS

### Study design

A national survey was distributed by electronic mail to all participants through the Association of Upper Gastrointestinal Surgeons (AUGIS). Centers were identified based on whether they carried out major esophageal or gastric resections for malignancy. A total of 39 centers were contacted to complete the questionnaire. Respondents were asked to answer the questions of the survey to provide an overview of service provision in their center on patients undergoing surgical resection with or without neoadjuvant and adjuvant therapy, for esophageal and gastric cancers for all stages. Participants were also asked to answer several questions aimed at determining the variation in surveillance practices, factors influencing surveillance protocols, management of oligometastatic and isolated local recurrence, center characteristics as well as their perspectives on current postoperative surveillance. The complete questionnaire and the list of participating centers are provided as a supplementary file (Supplementary file 1).

### Data collection and analysis

Data were collected on the Google survey platform. Briefly, the survey has three components. The first component collected data on the characteristics of the responders and their hospitals to describe the centers, specifically on the number of resections performed to determine if it was a high-volume center and number of surgeons performing the operations in each center to determine operative capacity and workload. The second section evaluated the surveillance protocols in the respective centers, including whether routine follow-up was undertaken; nature of the protocol (standardized vs. tailored to patient); general timeframe for the first clinical postoperative review; investigations carried out at interview and the specific personnel involved in follow-ups. The third section was aimed at collating the opinions on postoperative surveillance from surgeons performing these procedures at the highest volumes. These questions were aimed at both current surveillance patters as well as how they can be modified in the future; cost effectiveness; impact on patient quality of life and survival outcomes. All data are expressed as percentages where proportions or frequencies are reported.

## RESULTS

### Characteristics of survey responders

The characteristics of participating centers are shown in Table 1. Of the 39 centers approached to complete the survey, 35 respondents from 27 centers (70% completion rate) consisting 28 consultants, 6 senior trainees and 1 specialist nurse had completed the questionnaire. We received two separate responses from five centers and four responses from 1 center. Of the survey responders, 80% (*n* = 28) were consultants; 17% (*n* = 6) were senior trainees in higher surgical training or fellows; and 1 was a specialist nurse. 77.1% (*n* = 22) of the participating centers undertook at least 60 resections per year, and only two centers performed less than 40 resections.

**Table 1:** Characteristic of centers/respondents

Characteristic of center/respondent				
Role of respondent	80% Consultant	17.1% Senior trainee	2.9% Specialist nurse	-
**Number of resections**	77.1% >60 Cases	11.4% 50–60 Cases	5.7% 40–50 Cases	-
**Number of attending surgeons**	7–8 Surgeons (12.2%)	5–6 Surgeons (39.4%)	3–4 Surgeons (35.4%)	1–2 Surgeons (6%)

### Pattern of surveillance protocols

From the survey results, all patients were followed up by a member of the surgical team in the in the outpatient department or clinic. Most commonly, 45.7% of responders followed their patients up at 2–4 weeks; 34.3% at 0–2 weeks; 17.1% at 4–6 weeks; and 2.9% at greater than 6 weeks. Typically, they were seen by a surgeon (82.9%, *n* = 29). Three responders indicated that patients were only seen as required such as experiencing symptoms or other complications. Two centers routinely have their patients seen in a survivorship clinic, which can take place in another hospital different to site of surgery and hence by a team other than the primary surgical team. Twenty responders indicated having a specific postoperative surveillance protocol for their patients, and of these 31.4% (*n* = 11) reported having a standardized protocol for all patients, while 25.7% (*n* = 9) reported having a tailored protocol depending on what the patient required; 42.8% (*n* = 15) responded not having any specific routine postoperative surveillance protocols, of which nine undertook surveillance if indicated depending on clinician and patient factors, while 17.1% (*n* = 6) did not carry out routine surveillance for asymptomatic patients. Additional forms of follow-up was reported by most centers; 75% (*n* = 24) involved auxiliary follow-up by a cancer specialist nurse or a cancer coordinator; 21.9% by a member of radiation or medical oncology and 59.4% (*n* = 19) by the nutrition team; 53.1% of these follow-ups were also telephone follow-ups. There was concordance in the pattern of surveillance protocol between individuals from the same center, exception one case where one response indicated standardized protocol for all patients, while the second response indicated that surveillance was not undertaken for asymptomatic patients.

### Investigations in surveillance protocols

**Fig. 1:**
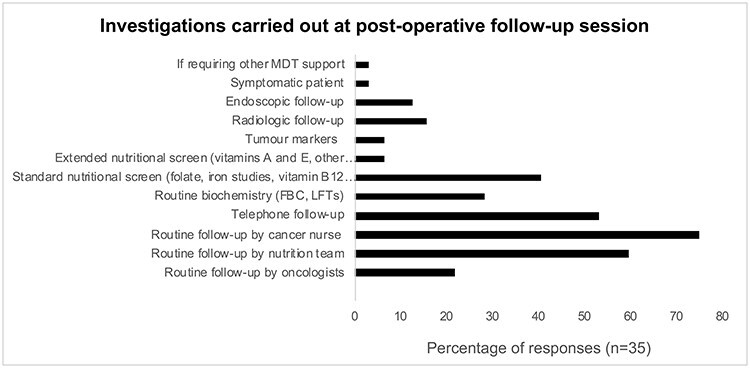
Investigations carried out at postoperative follow-up session.

**Fig. 2:**
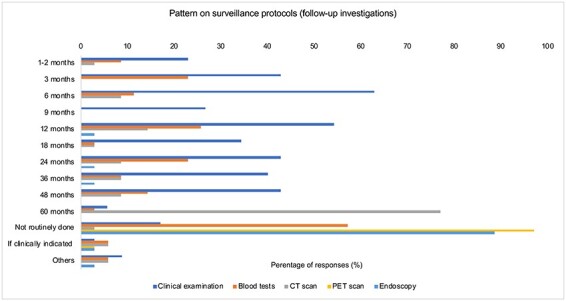
Pattern on surveillance protocols (follow-up investigations).

The practice of routine investigations varied between centers (Figs 1 and 2). 28.1% (*n* = 9) of centers performed routine blood tests, specifically biochemistry while 40.6% (*n* = 13) receive a standard nutritional screen including B12, folate and iron levels. In two centers, an extended nutritional screen evaluating vitamin A, vitamin E and micronutrient levels are also routinely performed. About, 6.3% (*n* = 2) of centers check levels of tumor markers such as CEA and CA19-9, while the remainder did not opt for this unless necessary (high levels pre-operatively or clinically indicated); 15.6% (*n* = 5) also provide routine radiological follow-up, while 12.5% (*n* = 4) provide routine endoscopic follow-up. Follow-up typically entails an update from the patient on their health status, and clinical examination, blood tests, imaging and/or endoscopy. Clinical examination was commonly performed at 6 (62.9%, *n* = 22) and 12 months (54.3%, *n* = 19). 17.1% (*n* = 6) reported they do not routinely perform a clinical examination. In contrast, 57.1% (*n* = 20) reported that they do not routinely arrange for blood tests at follow-up. Of the remainder, most performed blood tests at 3 (22.9%, *n* = 8), 12 (25.7%, *n* = 9) and 24 months (22.9%, *n* = 8).

**Fig. 3:**
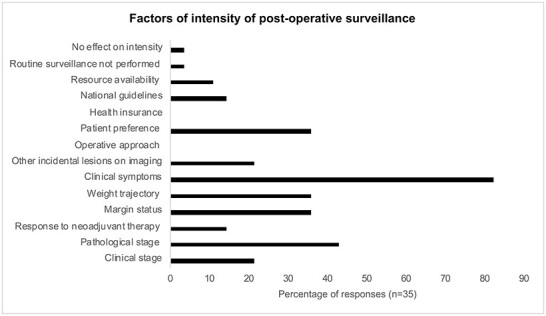
Factors of intensity of postoperative surveillance.

Similarly, 77.1% (*n* = 27) do not routinely request CT imaging, and when arranged, this was usually done at 12 months (14.3%, *n* = 5) and involved the chest, abdomen and pelvis. There was overwhelming agreement on not arranging a PET scan at follow-up (97.1%, *n* = 34) and the only other responder opted to request for it if clinically indicated. Similarly, 88.6% (*n* = 31) do not routinely carry out endoscopic investigations, while others may do it if clinically indicated, including if patients experienced any symptoms. When endoscopy is used, 81.8% reported the absence of a defined protocol for obtaining biopsies. Of the 18.2% (*n* = 4) who reported a defined protocol, three reported attaining targeted biopsies depending on the results of endoscopy. Additional investigations carried out included chromoendoscopy (*n* = 4, 66.7%) and endoscopic ultrasound with/without fine needle aspiration (*n* = 2, 33.3%). The most significant factor affecting the intensity of pre-operative surveillance was clinical presentation (82.1%, *n* = 23), followed by pathologic staging (42.9%, *n* = 12), margin status (35.7%, *n* = 10), weight trajectory (35.7%, *n* = 10) and patient preference (35.7%, *n* = 10) (Fig. 3). Of note, we had received responses from different individuals in the same center on six occasions, and there was no difference in surveillance patterns within centers.

### Perspectives on surveillance protocols

The extent of agreement on statements related to postoperative surveillance are shown in Table 2 and figures. Only 31.4% (*n* = 11) agreed that intensive surveillance may improve overall survival through earlier detection of local recurrence and/or oligometastatic disease and/or earlier treatment initiation, while 34.3% were not in favor of intensive surveillance. Similarly, 57.1% (*n* = 20) agreed that intensity/modality of oncologic surveillance protocols is unlikely to impact survival outcome in esophageal cancer, and 57.2% (*n* = 20) of responders felt that more intensive surveillance is not necessarily associated with reduced patient anxiety while 48.6% (*n* = 17) felt intensive surveillance will, in fact, increase patient anxiety. However, there was a general consensus that postoperative surveillance should be tailored to recurrence risk (62.9% agreement); patient preference (60% agreement); and patient factors such as age or comorbidities (65.7% agreement) but not type of treatment (surgery alone vs. multimodal therapy) (26.5% agreement); histology (adenocarcinoma vs. squamous cell carcinoma) (37.2% agreement); presence of background Barrett’s esophagus (31.4% agreement); tumor location (20.6% agreement); and Siewert/AEG classification (11.5% agreement). Only 17.2% (*n* = 6) felt that postoperative surveillance protocols will be cost-effective. Given the above variation in opinions, there was a general willingness (5.7% disagreement) to participate in a randomized controlled trial evaluating the prognostic value of intensive surveillance after esophageal and gastric cancer resections.

**Table 2:** Extent of agreement on statements related to postoperative surveillance

Survey statement	Percentage of respondents				
	Strongly agree	Agree	Neutral	Disagree	Strongly disagree
Intense surveillance improves overall survival	2.9	25.7	34.3	31.4	5.7
Intensity/modality of oncologic surveillance protocols is unlikely to impact survival outcome in esophageal cancer	5.7	51.4	28.6	11.4	2.9
Intensive surveillance is associated with reduced patient anxiety	5.7	2.9	34.3	48.6	8.6
Intensive surveillance is associated with increased patient anxiety	5.7	42.9	40	11.4	
Postoperative surveillance should be tailored to recurrence risk	8.6	54.3	14.3	20	2.9
Different surveillance protocols applied for patients on different therapy types	-	47.1	26.5	26.5	-
Different postoperative surveillance protocols should be applied for patients with adenocarcinoma vs. squamous cell carcinoma		34.3	11.4	51.4	2.9
The presence of background Barrett’s at diagnosis should influence postoperative surveillance protocols		31.4	14.3	51.4	2.9
The tumor location should influence modality of surveillance		20.6	26.5	52.9	
The Siewert/AEG classification should influence modality of surveillance		8.6	20	68.6	2.9
Postoperative surveillance should be tailored to patient preference	11.4	48.6	20	17.1	2.9
Postoperative surveillance should be tailored to patient factors (age, comorbidity)	8.6	57.1	8.6	8.6	2.9
Postoperative surveillance is cost effective		8.6	51.4	31.4	8.6

## DISCUSSION

Overall, our study highlights that there is a paucity of standardized protocols for surveillance of patients in the short and long-term after surgery for esophageal and gastric cancers in the UK and Ireland. This is reflected in the variation in follow-up periods, frequencies, personnel involved in the follow-up process, investigations arranged for patients as well as general attitude towards surveillance between different centers at a national level. While there is an urgency for aggressively investigating patients who may have symptoms, there is very little intervention for the asymptomatic population, including clinical examination during consultation. The use of radiological or endoscopic investigations is also minimal in most centers albeit at present not supported by current NICE guidelines in asymptomatic patients. There is strong evidence demonstrating better short and long-term outcomes due to centralization of services for esophageal and gastric cancers, and this can partly be attributed to strict evidence-based standardized clinical care pathways.^16^^,^^17^ It would be reasonable to standardize the surveillance strategy for patients at risk of early or locoregional recurrence if other clinical and patient-related factors are also incorporated into stratified surveillance protocols with appropriate therapeutic strategies.

Our study also highlighted the dominant factors affecting the intensity of postoperative surveillance. The major factors, as expected, included clinical symptoms, pathological staging and clinical stage. Surprisingly, patient preference was ranked as a factor by only 35% of respondents. Similarly, only one center reported the inclusion of patient’s opinion on imaging at follow-up. In most other aspects of the cancer pathway, there is a huge emphasis on empowering the patient in a shared decision-making process.^18^ Although previous studies have highlighted that this may only cause unnecessary anxiety, the overall quality of life is unchanged given that patients often also worry about recurrence, and interval scans may not only reassure the patient but also detect asymptomatic recurrence. This is further supported by previous work from the Netherlands, where patients showed a strong preference for routine surveillance after esophagectomy, with 67% preferring imaging even if this approach would not provide a survival benefit.^19^ A similar trend has been noted in other cancers as well.^20^ In the context of a shift towards patient-centered provision of oncological services, patients need to be better engaged in planning their postoperative surveillance. This is also supported by the opinions of our survey participants.^18^

Our work also registers highly heterogeneous opinions regarding the relative merits and negatives of intensive surveillance. For example, most physicians were either doubtful or disagreeable on the cost-effectiveness of surveillance and feel further intense surveillance is unlikely to change the survival outcomes to a significant extent. However as treatments do improve for recurrent disease, the rationale for attempting to identify recurrence at an early loco-regional stage becomes increasing important.^21^^,^^22^ Follow-up is often intense in the first 12 months when most complications occur and subsequently routine investigations rarely changed clinical practice given that complications were either less frequent or management options were limited if a recurrence did occur. The vast majority were also doubtful of the issues raised in the question and were open to participating in more stringent prospective studies such as randomized controlled trials, which are necessary in light of conflicting evidence on the utility of postoperative surveillance.

Many centers use specialist auxiliary staff to provide a holistic service. This is best reflected by dietician clinics indicated in many centers that specifically target the nutritional status of the patient. These clinics also perform routine investigations at every follow-up, including standard biochemistry tests and extended nutritional screens. Given the complexity of upper GI cancers, the postoperative surveillance will require a multi-disciplinary team (MDT), if a standardized surveillance protocol were to be introduced. Further expert opinion is required to determine what specific teams will be required, what their roles will be and how they fit into the surveillance pathways. It is possible that a MDT discussion may be required similar to the pre-operative phase, and this links back to the cost-effectiveness of postoperative surveillance and raises a need for formal economic analyses as future work.

The strength of our study relies on it being a nationwide questionnaire involving all high-volume centers performing procedures for upper GI cancers within a centralized service, which adds to the generalizability of the data. It also captures variations in practice amongst different centers at both a geographical and center level. It is tailored to provide a snapshot of current clinical practice while also incorporating a summary of expert opinions from practicing surgeons on how surveillance should be performed. One limitation of this study is that it includes entries from a single respondent completing the questionnaire on behalf of a center, so while it can delineate variations in practice between centers, it may not accurately identify differences at a clinician level. While over 70% of responses stem from a high-volume center (>60 resections per year) as the UK has a centralized esophago-gastric cancer service. Thus this limits the external validity of the results to smaller volume centers, especially from an economic perspective. Furthermore, our survey does not provide the granularity to capture the impact of the histological subtype (EAC, ESCC or gastric cancer) or the surgical intervention performed on the surveillance strategy. Although this approach is consistent with current NICE guidelines, which group these cancers and their intervention under one category, we acknowledge that these are biologically distinct cancers and hence would behave differently.^15^ It would be interesting to link these with oncological and survival outcomes of patients, and whether there is a relationship between more standardized surveillance, and morbidity and mortality of patients. This is an area for future work, and the results can be useful in generating updated clinical guidelines for the surveillance of esophageal and gastric cancers.

## CONCLUSION

There is currently no evidence-based guidelines for postoperative surveillance after esophago-gastric resections in the UK and Ireland. Consequently, there is a significant variation in how patients are monitored after surgery. Amongst surgeons from high-volume centers, there is little consensus on what follow-up should involve in terms of time-frame, personnel involved and the components of follow-up, although there is agreement that patient preference and comorbidities are significant factors to consider in planning surveillance. Given the above, further prospective work specifically randomized controlled trials are necessary to link surveillance strategies to both survival outcomes and patient related outcomes on quality of life.
